# Combined intermittent hypobaric hypoxia and muscle electro-stimulation: a method to increase circulating progenitor cell concentration?

**DOI:** 10.1186/1479-5876-12-174

**Published:** 2014-06-19

**Authors:** Luisa Corral, Casimiro Javierre, Juan Blasi, Ginés Viscor, Antoni Ricart, Josep Lluis Ventura

**Affiliations:** 1 Intensive Care Unit of Bellvitge University Hospital and Department of Physiological Sciences II of University of Barcelona, Feixa Llarga s/n. L’Hospitalet de Llobregat-08907, Barcelona, Spain; 2 Department of Physiological Sciences II, University of Barcelona, Feixa Llarga s/n, L’Hospitalet de Llobregat-08907, Barcelona, Spain; 3 Department of Pathology and Experimental Therapeutics, University of Barcelona, Feixa Llarga s/n, L’Hospitalet de Llobregat-08907, Barcelona, Spain; 4 Department of Physiology and Immunology, University of Barcelona, Av. Diagonal, 645, Barcelona-8028, Barcelona, Spain; 5 Intensive Care Unit, Bellvitge University Hospital, Feixa Llarga s/n, L’Hospitalet de Llobregat-08907, Barcelona, Spain

**Keywords:** Hypoxia, Hypobaric, Muscle electro-stimulation, Progenitor cells

## Abstract

**Background:**

Our goal was to test whether short-term intermittent hypobaric hypoxia (IHH) at a level well tolerated by healthy humans could, in combination with muscle electro-stimulation (ME), mobilize circulating progenitor cells (CPC) and increase their concentration in peripheral circulation.

**Methods:**

Nine healthy male subjects were subjected, as the active group (HME), to a protocol involving IHH plus ME. IHH exposure consisted of four, three-hour sessions at a barometric pressure of 540 hPa (equivalent to an altitude of 5000 m). These sessions took place on four consecutive days. ME was applied in two separate 20-minute periods during each IHH session. Blood samples were obtained from an antecubital vein on three consecutive days immediately before the experiment, and then 24 h, 48 h, 4 days, 7 days and 14 days after the last day of hypoxic exposure. Four months later a control study was carried out involving seven of the original subjects (CG), who underwent the same protocol of blood samples but without receiving any special stimulus.

**Results:**

In comparison with the CG the HME group showed only a non-significant increase in the number of CPC CD34+ cells on the fourth day after the combined IHH and ME treatment.

**Conclusion:**

CPC levels oscillated across the study period and provide no firm evidence to support an increased CPC count after IHH plus ME, although it is not possible to know if this slight increase observed is physiologically relevant. Further studies are required to understand CPC dynamics and the physiology and physiopathology of the hypoxic stimulus.

## Background

Human circulating progenitor cells (CPC) have generally been defined as circulating cells that express a variety of cell surface markers similar to those expressed by vascular endothelial cells, that adhere to endothelium at sites of hypoxia/ischemia, and which participate in new vessel formation, contributing to the maintenance of endothelial function and organ perfusion through mechanisms that range from endothelial repair to neovasculogenesis [1-3]. Several studies have found that elevated concentrations of CPC correlate with better clinical outcomes [4]. An increase in CPC has been observed after various events including myocardial infarction [5], dilated myocardiopathy [6], cardiac surgery with cardiopulmonary bypass [7], 12 weeks of physical exercise [8,9], menstruation [10], cessation of smoking [11] and traumatic brain injury (TBI) [12], as well as in animal or human cells subjected to deep hypoxia conditions in vitro [13-16].

Hypoxia enhances proliferation and tissue formation derived from mesenchymal stem cells in human bone marrow [14]. Functional benefits of repetitive hypoxia have been explored not only for their therapeutic value in patients but also as regards performance improvement in athletes [17]. Above 2500 m of altitude, hypobaric hypoxia elicits different adaptive responses and may also cause certain diseases such as acute mountain sickness, an outcome that depends mainly on the ascent speed and the time spent at altitude [18]. However, when producing intermittent hypobaric hypoxia (IHH) in a hypobaric chamber it is possible to control the temporal parameters equivalent to ascent time, altitude time and descent time, thereby greatly reducing the risk of complications by limiting and tailoring the exposure time to each target and each patient’s tolerance.

Research also suggests that exercise may influence the mobilization of CPC, and probably tissue homing as well, both in healthy people [19-23] and in cardiovascular or renal patients [24-26]. Although the different forms of standard physical exercise may be difficult to implement under hypoxic conditions, muscle electro-stimulation (ME) is easier to apply and has been shown to be efficient in mimicking training effects and increasing CPC [27-29].

A recent study by our group suggested that a short protocol of exposure to IHH in a hypobaric chamber with simultaneous ME is able to increase the concentration of CPC in peripheral blood in humans [29]. This promising finding raises the hope that an increase in CPC could help in the recovery from many diseases. Previous studies have used a variety of protocols to assess CPC mobilization [17-26], but their results are not conclusive. A better understanding of the issue in healthy humans may help guide the design of clinical applications and thus aid recovery from a variety of diseases.

The present research sought to reproduce our previous study with a larger sample size, more post-stimulus blood samples and the inclusion of a control group, the aim being to determine whether short-term IHH at a level well tolerated by healthy humans could, in combination with ME, increase CPC.

## Methods

Nine healthy males were subjected to a protocol involving IHH plus ME, hypoxic muscle electro-stimulation as the active group (HME). Their median age was 29 years [interquartile range (IQR) 9]. Six of them performed regular athletic exercise (three fewer than 3 times/week and three more than 3 times/week) and one was a smoker. Median height was 176 cm (IQR 11.5), mean weight was 80 kg (IQR 14) and median BMI was 25 (IQR 3).

IHH exposure consisted of four, three-hour sessions at a barometric pressure of 540 hPa (equivalent to an altitude of 5000 m). These sessions were conducted on four consecutive days in the hypobaric chamber of the University of Barcelona at temperature between 24.1 and 26.7°C and relative humidity between 32 and 39%. During the IHH sessions ME was applied using the Compex Vitality® vascular and capillarization program, with electrodes being fixed to the quadriceps and abdominal muscles [30]. ME was applied at the maximal tolerated intensity (regulated individually by each experimental subject) in two separate 20-minute periods during each IHH session.

Blood samples were obtained from an antecubital vein on three consecutive days immediately before the experiment, and then 24 h, 48 h, 4 days, 7 days and 14 days after the last day of hypoxic exposure.

A control study was conducted four months later with seven of these same participants (CG). On this occasion they did not receive any special stimulus, neither IHH nor ME was used, but blood samples were obtained according to the same schedule and on the same days of the week.

### Blood sampling, CD34 staining and flow cytometry assay

All blood samples were obtained in the morning and following the same extraction methodology, as detailed below. Peripheral blood samples were collected by puncture of an antecubital vein and deposited into tubes treated with 0.34 M di-potassium ethylenediaminetetraacetic acid anticoagulant. All samples were stored at room temperature and processed within six hours of arrival at the laboratory. Samples were incubated for cytometric absolute count with anti-human fluorescein isothiocyanate (FITC)-conjugated CD45 monoclonal antibody (BD Bioscience) and anti-human phycoerythrin (PE)-conjugated anti-CD34 (BD Bioscience) for 15 min at room temperature. Red blood cells were lysed with 1 ml of quick lysis solution (CYT-QL-1, Cytognos) for 15 min at room temperature. Samples were incubated under dark conditions and analysed immediately. To ensure accuracy, reverse pipetting was used to dispense the volumes.

A single-platform protocol with Perfect-Count microspheres (CYT-PCM-50, Cytognos, Salamanca, Spain) was used according to manufacturer’s instructions. The Perfect-Count microsphere system contains two different fluorospheres in a known proportion (A and B beads), thereby assuring the accuracy of the assay by verifying the proportion of both types of beads. Known volumes (50 μL) of Perfect-Count microspheres were added to the same known volume (50 μL) of stained blood using a lyse-no-wash technique, with the beads being counted along with cells. Cell viability was measured by staining the samples with the vital dye 7-aminoactinomycin D (7-AAD), as proposed by ISHAGE guidelines [31]. Samples were analysed on a FACSCanto II flow cytometer (BD Biosciences) with a 488-nm argon laser and DIVA 6.1.3 software (BD Bioscience). The gating strategy was in accordance with ISHAGE guidelines [31].

All aspects of the study were approved by our Bellvitge University Hospital’s Research Ethics Committee, and informed consent was obtained from all participants.

### Statistical analysis

Data are expressed as mean, median, standard deviation and interquartile range, as appropriate. Continuous variables were compared between groups by means of analysis of variance (ANOVA) or the Mann-Whitney U test, as appropriate. The Wilcoxon signed-rank test and the Friedman test for repeated measures were also used. All tests were performed using SPSS v.13. Statistical significance was set at p < .05.

## Results

**Figure 1:**
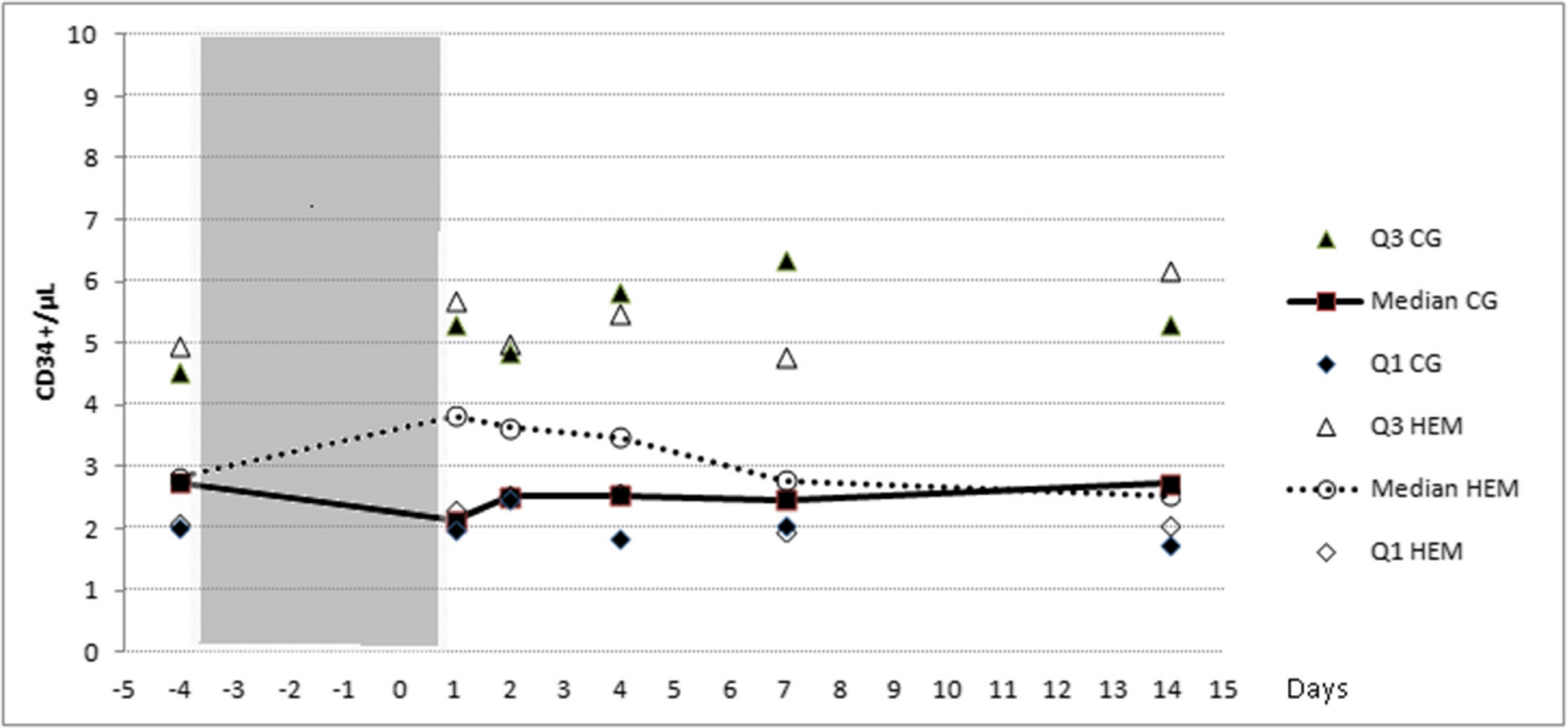
**Median and IQR values of CPC CD34+/μL in the HEM and control groups (CG).** Days of hypobaric chamber are shaded in grey. Q1 HEM: 1^st^ quartile of the HEM group, Q3 HEM: 3^rd^ quartile of the HEM group, Q1 CG: 1^st^ quartile of the CG, Q3 CG: 3^rd^ quartile of the CG.

**Figure 2:**
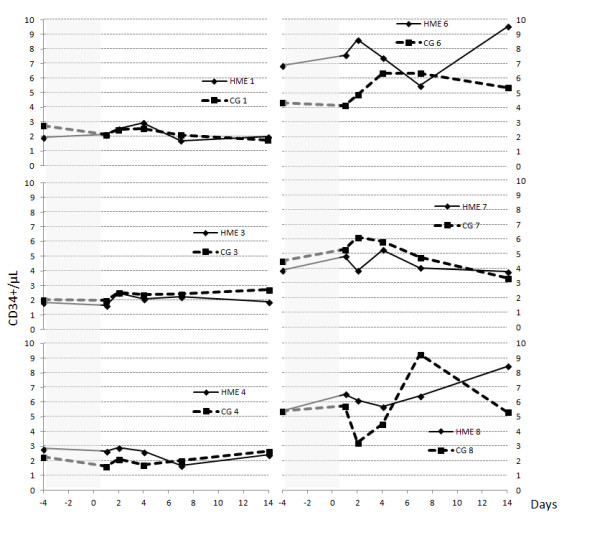
**Graphs of individual CPC values in six paired subjects of HEM and CG.** Days of hypobaric chamber are shaded in grey. HME: continuous line in each subject and CG discontinuous line in each subject.

The initial values of CPC CD34+/μL ranged from 1.54 to 5.41 in the control group (CG) and from 1.83 to 6.88 in the active group (HME). Compared with the CG the HME group showed a very slight increase in the number of CPC on the fourth day after IHH and ME, but this difference was not significant. Figure 1 shows the absolute values of CPC in the HME and CG groups and Figure 2 shows in different graphs individual CPC values for the HEM and CG. There were no statistically significant differences in CPC count either within-subjects (Friedman test) or between groups (Mann-Whitney U test). Multiple comparisons were made by pairwise comparisons of the variables with the Wilcoxon signed-rank test: there was a statistically significant difference between CPC on the second day with respect to pre-intervention CPC (3.38 (IQ range 2.52-6.76) vs 3.35 (IQ range 1.92-5.78,) p = 0.046) and on the fourth day with respect to pre-intervention day in the HME group (4.10 (IQ range 2.51-6.12) cells/uL) vs 3.35 (IQ range 1.92-5.78), p = 0.046). There were no statistically significant differences in the control group; nor were the differences between the HME and the CG significant.

During hypobaric hypoxic periods at a simulated 5000 m altitude, SaO_2_ and heart rate (HR) were measured five times per session per participant with a pulse oximeter (180 measures). Mean SaO_2_ was 75.6% (SD 5.6) and mean HR was 89 beats per min (SD 15).

## Discussion

This study found no a significant increase in the number of CPC after IHH plus ME, as compared with a control group. This result contrasts with the findings of our previous study, in which IHH plus ME seemed able to increase the concentration of CPC in four healthy men aged around 50 years of age. In this previous experiment the CPC count increased from a median value of 0.95 cells · μL^-1^ (range: 0.5–2.1) to a median level of 6.65 cells · μL^-1^ (range: 3.7-10.7), this increase being highly significant [29]. As CPC levels appeared still to be increasing seven days after the last hypoxia session, it was not clear whether a plateau or maximum value had been reached.

The opposite results have been found in some studies using different protocols. Specifically, hypoxia decreased CPC count in two normobaric hypoxia studies involving a total of 20 subjects [32,33], and also in a hypobaric hypoxia study with 15 mountain trekkers [34]. Different types and intensities of physical exercise have also been shown to be capable of mobilizing CPC. Exercise increased CPC in rats [35] and humans [8,9,19,20,22,36], although one study of 20 healthy humans found no such effect [37].

Hypoxia with exercise increased CPC count in three studies [24,29,38] and improved haemodynamic and microvascular endothelial function in another [39], although it should be noted that the protocols applied were quite different. Ciulla et al. reported the case of one subject with increased CPC count following the high-altitude hypoxia and exercise oxygen demands of a trek in the Himalayas [38]. As already noted, Viscor et al. reported increased CPC after IHH and ME [29], and Wang et al. found that CPC count increased after five weeks of normobaric hypoxic training on a bicycle ergometer at 60% VO_2max_[24]. Wang et al applied a different protocol comparing normobaric hypoxia of 15% and 21%, with five days per week of cycling training for five weeks, that is, a total of 25 days of stimulus. On average they found increased CPC, but the responses shown in Figure 2 ranged widely: some subjects presented increases, others decreases, and still others no alteration. For their part, Shatilo et al. showed how ten days of normobaric isocapnic hypoxia improved haemodynamic and microvascular endothelial function in untrained subjects compared with trained healthy senior men [39].

The small samples used in these different studies make it difficult to draw any firm conclusions, since there are many factors that might influence the results. It is worth noting, however, that whereas many types of standard physical exercise can be difficult to implement in the context of hypoxic procedures or when osteoarticular conditions are present, ME is easier to apply and has been shown to be efficient in reproducing training effects [27,28]. That said, muscle adaptations to electro-stimulation could be different [40], and although normobaric and hypobaric hypoxia are similar, they can provoke different physiological responses [41,42].

Inter-individual CPC variability [37,43] and technical variability in CPC enumeration [44] may also influence the measured response. Compared with the CG, the HME group showed a very slight increase in CPC on the fourth day after IHH and ME. The increase was smaller than physiological inter- and intra-individual variations. However, wide variations have been shown in other studies [24,37,43,44]; therefore, we think that it is very important to establish the normal population values, daily spontaneous and/or stimulated physiological changes, the possible different rhythms in healthy people, and the possible variations between responders and non-responders.

Age has been related with less capacity to produce neurogenesis [45] and with different progenitor cell responses [9], although healthy elderly subjects have been shown to be capable of improving CPC clonogenic and migratory capacity after exercise [9]. In this regard, it should be noted that the subjects recruited for the present research were much younger than the four subjects included in our previous study [29]. One possible explanation is that IHH and ME may be more aggressive stimuli in older than for younger subjects. Some studies have reported a time-dependent increase in CPC count after a cycle incremental exercise test under normoxic or hypoxic conditions, increasing 10 min after exercise [21], during 240 min of strenuous exercise [22] and after a marathon race or 1500m field test [19].

Intermittent hypoxia (IH) has been shown to be physiologically beneficial in various groups of healthy and ill people [39,46,47]. IH exposure sessions have been used to improve physical condition and to treat several illnesses. Most of these interventions have been derived from studies carried out in the former Soviet Union, although this was done without a clear understanding of the holistic effects [33]. Hypoxia exposure has also been combined with normal athletic training in a variety of ways [48]. More specifically, IHH exposure has been shown to be an efficient stimulus for eliciting different adaptive responses so as to improve peripheral oxygen supply [49,50]. Serebrovskaya et al. reported similar results with a normobaric hypoxia exposure programme that was able to enhance innate immunity by mobilizing progenitor cells, activating neutrophils and by increasing circulating complement and immunoglobulins [33]. Serebroskaya et al. found an increase in hematopoietic stem and progenitor cells (HSPC) during the final 15 to 20 seconds of a 5-minute normobaric hypoxia bout, which was maintained 15 minutes after four bouts of hypoxia and returned to normal 30 minutes later. After a 14-day program of IHT (intermittent hypoxia treatment), the HSPC count had not changed one day after completion of the program, but had fallen by the seventh day.

The present study does have certain limitations, notably the small sample size and the variability of the techniques used to detect CPC in blood [44]. It is likely that the schedule used for blood sampling also made it difficult to detect rapid and brief transitory changes in CPC, but this study design was based on previous results obtained by our group in which the CPC increase was maintained for seven days. The aim was to find a short stimulus able to maintain the CPC increase over time in order to aid recovery from a variety of diseases. Some studies have reported a time-dependent increase in CPC count after exercise and/or hypoxia [19,22].

## Conclusion

Taking these limitations into account, the results of the present pilot study fail to confirm the significant increase in CPC after IHH plus ME previously reported by our group. It is not possible to know whether the slight increase observed is physiologically relevant, or whether there was also increase at other points after exercise. These results are controversial, and further studies including a larger number of subjects and more exhaustive blood sampling times and technique are required to clarify the possible effect of IHH and ME on the release of progenitor cells. This is particularly important with regard to their possible physiological or pathophysiological significance.

## Competing interests

The authors declare that they have no conflict of interest to disclose.

## Authors’ contributions

All six authors have contributed to the study in accordance with the international consensus on authorship, and they have approved the final draft submitted. LC contributed to the design and implementation of the study, to data collection and analysis, and to manuscript writing. CJ contributed to the conception and design of the study, to its implementation, to data collection and analysis, and to manuscript writing. JB contributed to implementation of the study, performed the measurements of circulating progenitor cells, and contributed to data analysis and manuscript writing. GV contributed to the conception and design of the study, to its implementation, to data collection and analysis, and to manuscript writing. AR contributed to the design and implementation of the study and to manuscript writing. JV made a significant contribution to the conception and design of the study, as well as to data analysis and manuscript writing. All authors read and approved the final manuscript.
